# Extraversion Is Linked to Volume of the Orbitofrontal Cortex and Amygdala

**DOI:** 10.1371/journal.pone.0028421

**Published:** 2011-12-09

**Authors:** Henk Cremers, Marie-José van Tol, Karin Roelofs, Andre Aleman, Frans G. Zitman, Mark A. van Buchem, Dick J. Veltman, Nic J. A. van der Wee

**Affiliations:** 1 Institute for Psychological Research, Clinical, Health and Neuropsychology Unit, Leiden University, Leiden, The Netherlands; 2 Leiden Institute for Brain and Cognition, Leiden University, Leiden, The Netherlands; 3 Department of Psychiatry, Leiden University Medical Center, Leiden, The Netherlands; 4 BCN Neuroimaging Center, University Medical Center Groningen, University of Groningen, Groningen, The Netherlands; 5 Department of Radiology, Leiden University Medical Center, Leiden, The Netherlands; 6 Department of Psychiatry, VU University Medical Center, Amsterdam, The Netherlands; French National Centre for Scientific Research, France

## Abstract

Neuroticism and extraversion are personality factors associated with the vulnerability for developing depression and anxiety disorders, and are possibly differentially related to brain structures implicated in the processing of emotional information and the generation of mood states. To date, studies on brain morphology mainly focused on neuroticism, a dimension primarily related to negative affect, yielding conflicting findings concerning the association with personality, partially due to methodological issues and variable population samples under study. Recently, extraversion, a dimension primarily related to positive affect, has been repeatedly inversely related to with symptoms of depression and anxiety disorders. In the present study, high resolution structural T1-weighted MR images of 65 healthy adults were processed using an optimized Voxel Based Morphometry (VBM) approach. Multiple regression analyses were performed to test for associations of neuroticism and extraversion with prefrontal and subcortical volumes. Orbitofrontal and right amygdala volume were both positively related to extraversion. Extraversion was differentially related to volume of the anterior cingulate cortex in males (positive) and females (negative). Neuroticism scores did not significantly correlate with these brain regions. As extraversion is regarded a protective factor for developing anxiety disorders and depression and has been related to the generation of positive affect, the present results indicate that the reduced likelihood of developing affective disorders in individuals high on extraversion is related to modulation of emotion processing through the orbitofrontal cortex and the amygdala.

## Introduction

Neuroticism and extraversion are personality factors that have been directly linked to emotional states: neuroticism has been associated with susceptibility to negative emotional states, whereas extraversion has been linked to susceptibility to positive emotional states [1]. Also, neuroticism correlates positively, whereas extraversion correlates negatively, with (subsyndromal) symptoms of depression and anxiety in the general population [2]. Other personality traits, such as agreeableness, openness to experiences, and conscientiousness, have been proposed to play a more indirect role in influencing affective states [1]. Consequently, neuroticism and extraversion may respectively put one at risk for [3], [4] or protect one against [3] development of affective disorders such as depression, panic disorder and social anxiety disorder.

More insight into the relation between personality and brain regions associated with emotion processing and regulation may help to illuminate how personality is involved in the processing of emotional information, and hence, in vulnerability to mood and anxiety disorders. On a functional level, it has previously been shown that neuroticism modulates neural activity in prefrontal and subcortical brain regions related to affective processing [5], [6]. Neuroticism has been related to more amygdala activation in response to distracting negative facial expressions during a cognitive task [7], and to less anterior cingulate cortex (ACC)–amygdala connectivity during processing of negative emotional facial expressions [5]. Extraversion on the other hand, was found to be positively associated with amygdala activation in response to happy faces [8]. Together, these findings indicate that core brain structures involved in emotion processing (e.g. the amygdala, the anterior cingulate gyrus, and the ventral part of the prefrontal cortex including the orbitofrontal cortex (OFC)) may play a role in the relation between personality, the processing of emotional information, and the production of mood states [9].

Structural variation might underlie the relation between personality and activity in brain regions related to emotion perception and appraisal. In an adolescent sample, a positive association between extraversion and (medial) prefrontal volume [10] has been observed, which has also been demonstrated in adults [11], although not consistently [12]. Therefore, the relation between brain volume and affect-related personality traits remains unclear. Inconsistencies in reported findings may be due to methodological and technical issues, such as the use of different segmentation and normalization strategies (e.g. analyses based on modulated vs unmodulated images [13], not accounting for total brain volume [14], or to sample characteristics and processes of brain maturation). For example, Jackson et al., 2009 [15], and Wright et al., 2007 [16] studied the relation between neuroticism and regional brain volume in an elderly population, whereas Blankstein et al., 2009 [10] included an adolescent sample. As aging is an important predictor of regional brain volume, and because the process of aging (including brain maturation) has been shown to interact with personality [15], it is possible that the conflicting finding may depend on the age range of the sample. It is therefore necessary to further elucidate the complex relation between affect-related personality traits and regional brain volume in an adult sample, controlling for these important factors (i.e. age, total brain volume and modulation) but also testing for possible interaction effect with sex, as Blankstein et al. 2009 [10] suggested that personality differentially affects regional brain volume in male and female adolescents.

In the present study, we used an optimized VBM approach to investigate the relationship between neuroticism, extraversion, and regional brain volume in a large sample of healthy adults. We focused on brain regions involved in the initial processing of emotional information (amygdala) and on regions related to the appraisal and decision making influence of emotional information, ACC and orbitofrontal cortex [16]. As such, we aimed to identify neuro-anatomical substrates associated with affect-related personality traits.

## Methods

### Ethics Statement

The study was carried out in accordance with the Declaration of Helsinki. Also, the Ethical Review Boards of the Leiden University Medical Center (LUMC), Academic Medical Center (AMC), University of Amsterdam, and University Medical Center Groningen (UMCG) approved this study. All participants provided written informed consent after complete description of the study.

### Participants

Sixty-five healthy participants were selected from the NESDA (Netherlands Study of Depression and Anxiety) neuroimaging study [17]. The MRI main sample is described in detail elsewhere [18]. Exclusion criteria for the current sample were: 1) a history of or current DSM IV axis I pathology, 2) taking any psychoactive drugs, 3) the presence or history of major internal or neurological disorder, 4) dependency or recent abuse (past year) of alcohol and/or drugs, 5) hypertension 6) general MR-contraindications.

### Personality questionnaire

To asses personality traits, all participants completed the NEO Five Factor Inventory [19].

### Image acquisition

Imaging data were acquired using Philips 3T MR-systems (Best, The Netherlands) located at the Leiden University Medical Center (LUMC), Academic Medical Center (AMC), University of Amsterdam, and University Medical Center Groningen (UMCG), equipped with SENSE-8 (LUMC and UMCG) and SENSE-6 (AMC) channel head coils, respectively. For each subject, anatomical images were obtained using a sagittal 3D gradient-echo T1-weighted sequence (TR = 9 ms, TE = 3.5 ms; matrix 256×256; voxel size: 1×1×1 mm; 170 slices).

### Data analysis

VBM following the Diffeomorphic Anatomical Registration Through Exponentiated Lie (DARTEL) algebra software [20] implemented in Matlab 7.1.0 (The Matlab Inc, Natick, MA, http://www.mathworks.com/) was used. The preprocessing and masking procedure is described in detail elsewhere [18]. Briefly, after segmentation, data were registered, normalized, and modulated using the DARTEL pipeline. Grey matter (GM) images were normalized to Montreal Neurological Institute (MNI) space and smoothed at 8 mm full width at half maximum (FWHM). In the resulting images, each voxel represents an absolute amount of brain volume, equivalent to the brain volume per unit prior to normalization.

Next, we performed a multiple regression analysis with neuroticism and extraversion as independent variables and voxel-wise GM density maps as the dependent factor. In addition, sex×personality interaction terms were calculated by setting up a flexible factorial design. In each model, age, scan center, and GM total volumes were entered as nuisance variables. Based on the literature on emotion processing in affective disorders [21], we set the following regions of interest (ROI): amygdala, orbitofrontal cortex (OFC), anterior cingulate cortex (ACC). A family wise error (FWE) at *p*<.05 correction for the spatial extent of the ROIs (small volume corrections) was applied. The ROIs were defined by the Automated Anatomical Labeling (AAL) templates implemented in the Wake Forest University School of Medicine (WFU) pickatlas [22]. For regions other than the ROIs, a voxel level threshold of *p*<.05 whole brain FWE corrected was set *a priori*. For completeness, explorative analysis are performed at an uncorrected threshold of p<.001, with a spatial cluster extent of 25 contiguous voxels. Demographic and clinical data were analyzed with SPSS 16.0 (http://www-01.ibm.com/software/analytics/spss/) and significance was set at *p*<.05.

## Results

### Sample characteristics and personality scores

The age range of the 65 participants (42 females) was: 21–56, Mean (M) = 40.5, standard deviation (sd) = 9.7; years of education: M = 14.3, sd = 2.9. Neuroticism (range = 13–36; M = 24.2, sd = 4.9) correlated negatively to extraversion (range 27–56; M = 43.7, sd = 6.2) (r = −0.38, *p* = .002). Neuroticism correlated negatively with total GM, whereas Extraversion correlated positively with total GM (Neuroticism: r = 0.29, *p* = .02; Extraversion: r = .29, *p* = .02). After partialling out variations in age and sex, only the correlation between Extraversion and GM total remained significant (Neuroticism: r_partial_ = −.17, *p* = .18; Extraversion: r_partial_ = .27, *p* = .03). Neither age nor years of education were significantly correlated with either neuroticism or extraversion (all *p*>.13), and no effect of sex was observed (F<1.74, *p*>.19)

### Personality and regional brain volume

**Figure 1:**
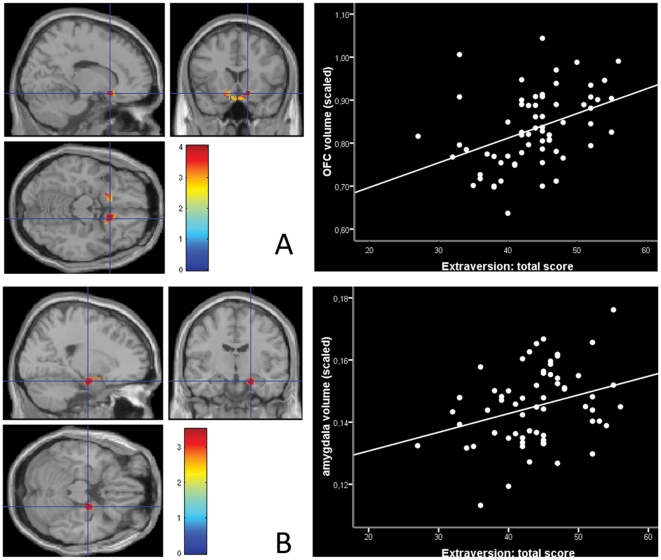
Correlations of Extraversion and volume in the OFC and amygdala. A) Positive correlation of extraversion and volume of the right orbital frontal gyrus scaled for total gray matter volume (r = .33; r_partial_ = .47 when neuroticism and age were partialled out) . B) Positive correlation of extraversion and right amygdala volume scaled for total gray matter volume (r = .31, r_partial_ = .39 when neuroticism and age were partialled out). Mean volume in ml of the significant voxels was extracted per subject and divided by total GM volume. In the correlation plots, mean volume of the significant amygdala and ofc region is depicted in scaled volume in ml * 10^−3^.

Volumes of both the right medial OFC (BA 11) (encompassing the rectal gyrus, the orbitofrontal region of Brodmann area 13 and the subgenual cingulate gyrus (BA 25)) and the right centro-medial amygdala were positively related with extraversion at the set threshold (*p*_FWE_<.05 corrected for extent of ROI). At *p*<.001 uncorrected, the positive correlation of extraversion was also observed in the left OFC. Neuroticism did not show a significant relation with regional brain volume in any of the ROIs. Adding the other three personality variables (openness, agreeableness, and conscientiousness) from the NEO FFI did not significantly change the results (OFC: Z = 3.28; amygdala, Z = 3.50). Results are shown in figure 1 and table 1. No significant whole brain FWE *p*<.05 corrected effects outside these ROIs were found. For completeness, positive and negative correlations of neuroticism and extraversion that were observed at *p*<.001, uncorrected with a spatial cluster extent of 25 contiguous voxels are listed in table 1 (*non-ROI effects*). However, these effects will not be further discussed, as these were not part of our *a priori* set regions of interest.

**Table 1:** VBM effects.

*positive correlations of extraversion*								
				MNI-coordinate				
R/L	region	BA	k	x	y	z	Z-score	r
**OFC**								
R	medial OFC, subgenual cingulate gyrus	11/25	329	13	16	14	3.77	.47
	medial OFC, rectal gyrus	11		2	22	18	3.54	.45
L	medial OFC, subcallosal gyrus	34	68	20	12	14	3.33	.42
**amygdala**								
R	amygdala, dorsal subdivision	n.a.	31	21	10	21	3.34	.42
***non-ROI effects***								
*R*	*superior parietal lobule*	*7*	*60*	*34*	*65*	*58*	*4.18*	
*L*	*cerebellar declive, posterior lobe*	*n.a.*	*65*	*56*	*65*	*29*	*3.93*	
*L*	*posterior cingulate gyrus*	*31*	*63*	*−7*	*41*	*27*	*3.60*	

*negative correlations of extraversion*								
				*MNI-coordinate*				
*R/L*	*region*	*BA*	*k*	*x*	*y*	*z*	*Z-score*	
*R*	*precentral gyrus*	*4*	*144*	*41*	*20*	*47*	*3.75*	
				*34*	*16*	*46*	*3.65*	
*L*	*calcarine sulcus*		*30*	*16*	*69*	*13*	*3.55*	
*L*	*inferior frontal gyrus*	*45*	*59*	*49*	*20*	*14*	*3.46*	
*L*	*superior parietal lobule*	*7*	*65*	*21*	*54*	*61*	*3.45*	

*positive correlations of neuroticism*								
				*MNI-coordinate*				
*R/L*	*region*	*BA*	*k*	*x*	*y*	*z*	*Z-score*	
*L*	*middle temporal gyrus*	*21*	*278*	*64*	*43*	*−2*	*3.86*	

*negative correlations of neuroticism*								
				*MNI-coordinate*				
*R/L*	*region*	*BA*	*k*	*x*	*y*	*z*	*Z-score*	
*R*	*supplemental motor area (SMA)*	*6*	*45*	*13*	*20*	*68*	*3.57*	

R/L: Right vs. Left hemisphere; BA: Brodmann area; k = clustersize at p<.001, uncorrected; r = correlation coefficient at peak voxel.

Extraversion×sex interaction analysis showed an effect in the pregenual ACC (MNI coordinate: [x = 3, y = 48, z = 7] (*p* = .01 FWE corrected for the volume of the bilateral ACC as defined by the AAL templates). Post-hoc analysis showed that males displayed a significant positive association with extraversion (β = .40, *p* = .008, 95% C.I.(B) = [0.005–0. 26], zero-order correlation: r = .43), with GM totals, neuroticism, age, and scan center added to the model, whereas the association was in the opposite direction in female (β = −.28, *p* = ,006, 95% C.I.(B) = [−0.24–−0. 004], zero-order correlation: r = .15). Sex by neuroticism interactions were not observed in any other region of interest.

## Discussion

In the present report, we used an optimized VBM approach to test for personality related variations in regional brain volume in an adult sample. While controlling for age, sex and total GM we demonstrated positive correlations between extraversion and regional brain volume in the medial OFC and centro-medial amygdala. This result confirms the role of the OFC in personality, a region that was already associated with personality changes in the case report of Phineas Gage [23]. We also found a positive correlation between extraversion and total gray matter volume. However, we did not find strong structural correlates of neuroticism.

The observation that extraversion correlated positively with volume of both the medial OFC (extending into subgenual ACC area 25) and the amygdala is of interest because of the role these regions play in affective processing [24], [25]. The medial OFC has often been associated with controlling reward and punishment related behavior, emotion regulation, approach related behaviour [26] and decision making [27], [28], and has projections to visceral control structures, such as the ventral striatum, amygdala, hypothalamus, periaqueductal grey, and hippocampus: regions that are critical in modulating behavior and emotional expression [26], [27], [29]. The amygdala has a well-documented role in emotion processing and has bi-directional connections with the medial OFC [27]. Interestingly, a positive correlation of OFC thickness with extraversion and fear extinction has been previously described [30]. In another study, humor-driven activation, reflecting hedonic capacity, was found to be positively correlated with extraversion in the right medial OFC [9]. Moreover, in the amygdala, activation during happy face viewing was found to be positively related to extraversion [6]. Hence, the increased volumes of medial OFC and amygdala may play a role in the increased sensitivity to positive, pleasant information and (social) reward, and thus, the propensity to experience the positive affect which characterizes extraversion [3]. The nature of this role, however, awaits further elucidation, as relations between volume and function are not straightforward: increased volumes of GM in brain areas may be reflective of a number of processes involving, among others, glial cells, inhibitory or excitatory neurons and interneurons. However, these processes cannot as yet be assessed in vivo in humans.

Beside the overall positive correlation between OFC and amygdala volume and extraversion, we also found a sex×extraversion interaction in the ACC. Males showed a positive correlation between ACC volume and extraversion, while this correlation was negative in females. A similar effect in the medial prefrontal gyrus was previously shown in adolescents [10]. These findings could suggest that, in men, the ACC is included in the same extraversion mediated regulatory network as the amygdala and OFC, while this is not the case in females. This could imply that extraversion has a stronger protective effect in men than in women, in line with the well known observation that men are less susceptible to affective disorders [31].

It is also interesting to note that our findings were mainly right-lateralized. In a study by Hasting et al, 2004 [32] it was found that non-medicated depressed patients showed smaller right amygdala volume than controls (an effect which was driven by the female participants). Since we found that low extraversion is linked to smaller right amygdala volume, our results can be considered in line with this study. Moreover, a meta-analysis on lateralization has found that the right amygdala is more involved in processing masked stimuli, whereas the left amygdala is more involved in processing stimuli which contain language [33]. Relating this finding to our data, it might suggest that extraversion is more strongly linked to the subconscious emotion-processing role of the amygdala. However, the lack of consensus regarding lateralization and amygdala function among functional MRI meta-analyses should be noted [34]–[36]. Moreover a positive correlation between the left amygdala and extraversion was also observed at a more liberal threshold (*p*<.005 uncorrected), and no formal interaction of extraversion×lateralization was observed in a repeated measures anova. Therefore, no strong statements on lateralization can be made. Furthermore the positive correlation between extraversion and right amygdala volume was localized in the central-medial amygdala, which contains (most of) the efferent connections from the amygdala to the brainstem and hypothalamus [27]. These connections are particularly important for fight/flight responses, and taken together, our findings might fit with the idea that extraversion is linked to moderating unconscious emotion processing and primary stress responses.

The present result with respect to extraversion is in line with findings of Blankstein and colleagues [10], who used a similar methodology (i.e. an optimized VBM approach) in an adolescent sample, and another study that showed a relation between extraversion and PFC volume in an adults sample [11]. However, also neuroticism has been related to OFC volume [12]. Accordingly, in the present study, we expected neuroticism to account for a substantial portion of the volumetric variation in regions associated with emotional perception and regulation. However, no such relation was observed. Instead, extraversion was found to be the main predictor of regional brain volume in affective brain regions. This discrepancy in findings might be due to methodological issues such as those outlined before, most importantly, this study used an optimized VBM method in a relatively large adult subject sample. Future research has to elucidate whether this lack of neuroticism - brain volume relations is a stable finding.

In the present study we examined structural correlates of extraversion and neuroticism in a cross-sectional design. Therefore, it is possible that the found correlates are not primary, but secondary to individual lifetime experiences. For instance, high extraversion is known to be associated with different lifetime experiences than low extraversion. Findings from the same NESDA study indicate that extraversion and negative life events mediate the course of depressive symptoms [37], suggesting that extraversion, also defined as the tendency to engage in reward-enhancing behavior, could influence the likelihood of experiencing positive life events or how certain life events are perceived. In addition, extraversion is argued to be a protective factor for dysthemia and social anxiety rather than other anxiety disorders [38]. Interactions between extraversion, lifetime experiences (both positive and negative) and brain structures should be investigated as this could shed light on the development of different psychiatric disorders.

Given the augmenting evidence for a significant relation between extraversion and OFC and amygdala volumes, future research should address structural and functional connectivity of the OFC and the amygdala, and further investigate the role of the pregenual ACC in this circuitry. This might provide more insight about the trajectory from health to psychopathology, and, in doing so, identify neuroanatomical markers which relates to one's risk of developing affective disorders.
